# Does Aerobic and Resistance Exercise Influence Episodic Memory through Unique Mechanisms?

**DOI:** 10.3390/brainsci10120913

**Published:** 2020-11-27

**Authors:** Paul D. Loprinzi, Damien Moore, Jeremy P. Loenneke

**Affiliations:** 1 Exercise & Memory Laboratory, Department of Health, Exercise Science and Recreation Management, The University of Mississippi, Oxford, MS 38677, USA; dcmoore3@go.olemiss.edu; 2 Kevser Ermin Applied Physiology Laboratory, Department of Health, Exercise Science and Recreation Management, The University of Mississippi, Oxford, MS 38677, USA; jploenne@olemiss.edu

**Keywords:** memory encoding, memory consolidation, neurotransmission, physical activity

## Abstract

Aerobic and resistance exercise (acute and chronic) independently and collectively induce beneficial responses in the brain that may influence memory function, including an increase in cerebral blood flow, neurogenesis, neuroelectrical alterations, and protein production. However, whether aerobic and resistance exercise improve memory via similar or distinct mechanisms has yet to be fully explained. Here, we review the unique influence of aerobic and resistance exercise on neural modulation, proteins, receptors, and ultimately, episodic memory. Resistance training may optimize neural communication, information processing and memory encoding by affecting the allocation of attentional resources. Moreover, resistance exercise can reduce inflammatory markers associated with neural communication while increasing peripheral and central BDNF (brain-derived neurotrophic factor) production. Aerobic training increases hippocampal levels of BDNF and TrkB (Tropomyosin receptor kinase B), protein kinases and glutamatergic proteins. Likewise, both aerobic and anaerobic exercise can increase CREB (cAMP response element-binding protein) phosphorylation. Thus, we suggest that aerobic and resistance exercise may influence episodic memory via similar and, potentially, distinct mechanisms.

## 1. Introduction

Both aerobic (physical activity that is maintained continuously, and rhythmic in nature [1]) and resistance exercise (the progressive use of resistance to increase one’s ability to exert or resist force [2]) are associated with numerous health outcomes, including reduced cancer incidence, decreased cardiovascular disease, and lower mortality risk [3,4]. Recent work also suggests that these two behaviors may play distinct roles on health. For example, research demonstrates that these two behaviors independently associate with health [3,4], with evidence also suggesting a potential synergistic effect of these behaviors on health [3,5].

In addition to these broad health outcomes, recent work has examined the potential effects of movement-related behaviors (e.g., aerobic vs. resistance) on cognitive function [6,7,8,9,10,11,12,13,14,15,16,17,18,19], including memory function. We do recognize, however, that additional research is needed that makes a side-to-side comparison between aerobic and resistance exercise on memory. Memory function can be broadly categorized into prospective (executing a plan in the future, such as remembering to call the doctor during a lunch break) and retrospective memory, with the latter including the recall of past events or episodes (i.e., episodic memory), often in a spatial and/or temporal context [20]. Further, other types of memory include, for example, implicit memory (driven by unconscious processes) and working memory, with the latter involving a transient storage of information while concurrently processing competing stimuli. The present paper is specifically focused on episodic memory.

The purpose of the present selective review is to briefly discuss past research evaluating independent and combined effects of acute and chronic aerobic and resistance exercise on episodic memory. We will then propose various explanations as to why these exercise modalities may, potentially, influence episodic memory via similar and distinct mechanistic pathways. Our intent was not to conduct a systematic review or meta-analysis, but rather to focus on studies evaluating the independent and additive effects of acute and chronic aerobic and resistance exercise on memory function and discuss its underlying mechanisms. Computerized searches occurred in PubMed, Google Scholar, and personal literature bases, with keywords including aerobic exercise, resistance exercise, and memory (and their combinations).

## 2. Acute Exercise Modality on Memory

In 2011, Labban and Etnier [21] evaluated the effects of 30 min of acute moderate-intensity aerobic cycling on memory performance (paragraph recall) in young adults. They demonstrated that exercising prior to memory encoding was effective in enhancing long-term memory performance (35-min retention interval). In 2016, Etnier et al. [22] evaluated the intensity-specific effects of aerobic (treadmill running for 30 min) exercise on memory function (list-learning paradigm) in young adults. Results showed that maximal acute exercise prior to memory encoding improved 24-h recognition memory and source memory relative to acute exercise below the ventilatory threshold. In 2018, Etnier et al. [23] showed that, among young adults, 30 min of moderate-intensity aerobic cycling, prior to memory encoding (list-learning paradigm), was effective in enhancing long-term (60 min and 24-h delay) memory recall, relative to a control condition. These memory enhancement effects of acute aerobic exercise have also been demonstrated in other related studies [24,25,26].

In addition to acute aerobic exercise, emerging research also demonstrates that acute resistance exercise may influence memory. Using a circuit-style acute resistance exercise paradigm, Loprinzi et al. [27] had participants complete five sets of exercises (e.g., push-ups, sit-ups, plank), with each set lasting 3-min, interspersed with a 1-min recovery between sets; the last set was completed to failure. Their results demonstrated that, among a young adult population, 15-min of acute resistance exercise was effective in enhancing episodic memory, specifically the “where” component of a spatial-temporal episodic memory task.

## 3. Chronic Exercise Modality on Memory

Iuliano et al. [28] conducted a 12-week randomized controlled study among 80 older adults (M_age_ = 67 years) who were randomized into a resistance, aerobic, postural, or a control group. The resistance exercise group performed progressive moderate-to-high loads of resistance exercises on isotonic machines involving six major muscle groups. During weeks 1–4, participants utilized a training load that was 60–70% of their one repetition maximum for three sets of twelve repetitions with three minutes of rest between each set. Subsequently, in weeks 5–8, the resistance load increased to 70–80% of their one repetition maximum for three sets of eight repetitions, with three minutes of rest between each set. Lastly, in weeks 9–12, the resistance load increased to 80–95% of their one repetition maximum for three sets of six repetitions, with three minutes of rest between each set. In a similar fashion, the aerobic group performed aerobic exercises (treadmill, bike, step-ergometers) of moderate-to-high intensity. The postural group performed various low-intensity postural and balance exercises. All intervention groups, compared to the control group, had less subjective memory complaints post-intervention, with no significant changes in objectively-measured memory performance between groups. Moreover, and although speculative, the reduction in memory complaint may have been due to exercise-induced improvements on mood, confidence, and self-efficacy.

In contrast to the findings above, longer exercise training interventions have shown mixed findings. Among a sample of older adults, Best et al. [29] showed that 52 weeks of resistance training (free weights, lunges), conducted twice a week (60 min per session), was effective in enhancing episodic memory from a list-learning paradigm. These findings are in partial agreement with the results of Jonasson et al. [30] who examined 6 months of stretching, aerobic, or resistance training in older sedentary adults. The aerobic intervention group demonstrated an improvement in cognition compared to the resistance group, with cognition defined by a host of cognitive tasks that included episodic memory, processing speed, executive function, and updating (*p* = 0.01).

Collectively, there is evidence demonstrating that acute and chronic aerobic and resistance exercise can, potentially, improve episodic memory function across differing ages of individuals. We now turn to whether combining both exercise modalities has an additive effect on memory. If so, this will suggest that these two exercise modalities may additively influence memory by activating the same mechanistic pathways and/or activating unique mechanistic pathways.

## 4. Combined Exercise Modality on Memory

Komulainen et al. [31] conducted a randomized controlled trial among older adults (*N* = 1335; 57–78 years). Participants were randomized into one of six groups (for a 2-year intervention) including a control (educational), aerobic exercise, resistance exercise, dietary, aerobic plus diet, or resistance plus diet. Of relevance here, the aerobic group exercised at 55–65% of VO_2max_ either at 5 × 90 min/week or 5 × 60 min/week (all exercises occurred on their own). The resistance exercise group, divided into two groups training either two or three sessions per week, engaged in an individualized strength-training program on their own. Each resistance training session involved the use of 10–14 muscle groups trained at an intensity of 60% of the one repetition maximum (two sets, 15 repetitions/set). These individuals were also instructed to engage in aerobic exercise of 150–180 min/week. At baseline and post-intervention, episodic memory was assessed from a word-list memory task. Results showed that improvements in maximal oxygen consumption in both the aerobic and resistance exercise groups, in addition to both the diet and combined aerobic exercise and diet groups, were associated with improved immediate episodic memory function compared to the control group. Nonetheless, these findings should be interpreted with caution. It is possible that the association of VO_2max_ improvement and episodic memory for the resistance training group is due to the aerobic exercise of 150–180 min/week that participants were instructed to engage in. Thus, the aerobic exercise that occurred in the resistance training group may be driving the association of cardiorespiratory improvement and episodic memory function. Importantly, although improvements in cardiorespiratory fitness in the intervention groups were associated with better memory performance, their primary analyses demonstrated that there no statistically significant differences in changes in memory between the study groups.

Aquino et al. [32] conducted a randomized controlled trial among 28 patients (M_age_ = 68 years) with chronic obstructive pulmonary disease (COPD) who were randomized into a high-intensity aerobic training group or a group that engaged in both high-intensity aerobic exercise and resistance exercise. Both groups exercised for 4-weeks and completed two daily training sessions, one session in the morning and the other in the afternoon. The aerobic group completed two 30-min daily aerobic sessions, while the combined group completed two 30-min daily sessions, with one being aerobic and the other involving resistance exercise. Both the aerobic (70–90% of HR_max_) and resistance exercise (70–90% of 1RM (repetition maximum)) sessions were progressive over the intervention period. Immediate and delayed memory was assessed from the Rey Auditory Verbal Learning Test. Delayed memory (long-term memory) significantly improved in both groups, but there was a greater magnitude of change in the combined training group.

Bossers et al. [33] conducted a 9-week randomized controlled trial among older adults (M_age_ = 85 years) with dementia. Participants were randomized into one of three groups, including a multimodal arm (two strength and two walking sessions per week), an aerobic arm (four walking sessions per week) and a social/control group (four social visits per week). The sessions were 30-min in duration, with an exercise intensity defined as a rating of perceived exertion between 12 and 15, which corresponded to a maximum heart rate between 50% and 85%. The strength sessions involved lower extremity exercises on large muscle groups (e.g., seated knee extension). Memory was assessed through multiple word-list and recognition tasks, including both short- and long-term memory assessments. Results showed that the combined exercise group, compared to the control group, improved visual memory and verbal memory. When comparing the combined exercise group to the aerobic only group, the combined group had slightly greater improvements in memory function.

## 5. Exercise Modality Mechanisms on Memory

Engaging in an exercise program that combines both aerobic and resistance exercise appears to have a greater effect when compared to an isolated exercise modality, even when matched for time [32]. Further, there is evidence that for the same type of exercise (e.g., running), modifying the modality (e.g., wheel running vs. treadmill running) may have unique neural adaptations in select brain regions [34]. As such, perhaps this additive effect is a result of these exercise behaviors activating both similar and unique mechanistic pathways.

**Figure 1:**
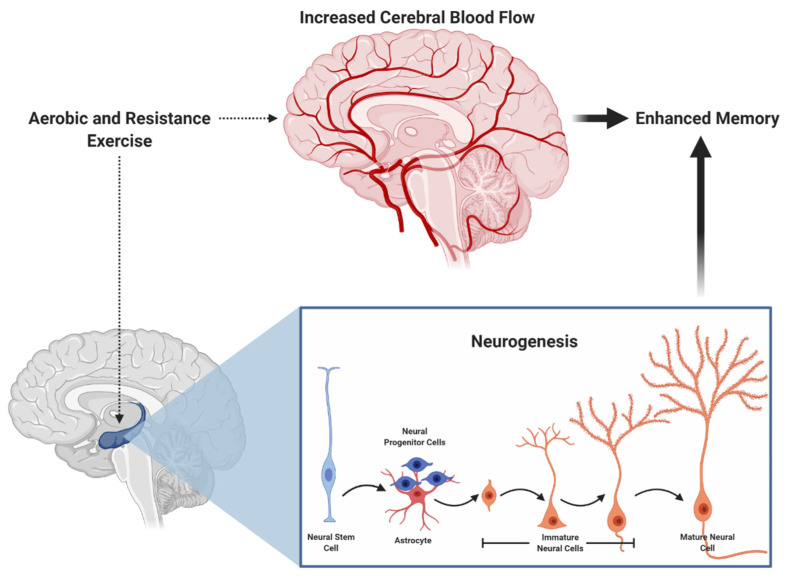
Aerobic and resistance exercise both induce hippocampal neurogenesis and increased cerebral blood, potentially leading to enhanced memory.

**Figure 2:**
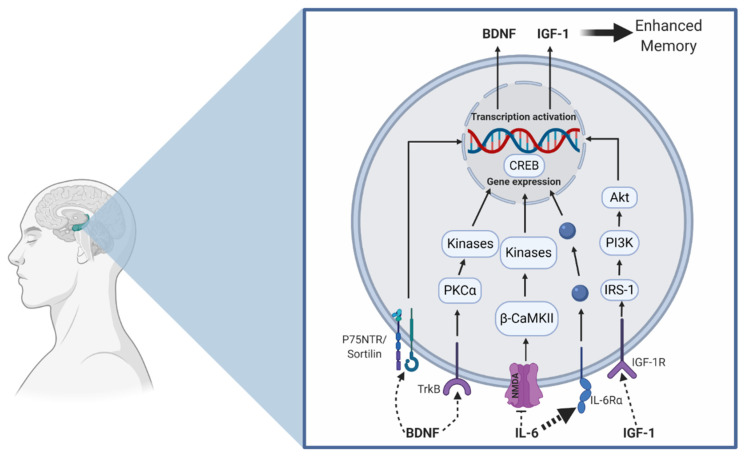
Hypothesized aerobic and resistance exercise model on improved hippocampal memory function. Aerobic exercise may increase proBDNF and hippocampal BDNF production, which potentially enhances CREB phosphorylation in addition to the upregulation of glutamatergic proteins (NMDA (N-Methyl-D-aspartate) receptor). Resistance exercise supports the reduction in IL-6 (**bold dash line**) in addition to increasing IGF-1 (Insulin-like growth factor-1) production. Consequently, IL-6 has been shown to inhibit NMDA activity. Furthermore, resistance exercise can increase the upregulation of the neurotrophic growth factor receptor (TrkB), in addition to protein kinase C alpha (PKCα). Ultimately, the activation of these pathways leads to gene expression and protein production, which has important implications on memory (see Table 1). This model depicts exercise-induced central production of these parameters, but we also recognize that some of these proteins are produced peripherally (from the skeletal muscle) and may cross the blood–brain-barrier to act centrally. The blue dots are a general representation of various proteins that act to facilitate gene expression.

Similarly, both aerobic and resistance exercise have been shown to favorably influence cerebral blood flow [35,36,37] and neurogenesis [38,39], which may subserve episodic memory function [40,41] (see Figure 1 for an illustration). More specifically, acute exercise may induce a transient increase in cerebral blood flow, whereas chronic exercise may increase capillarization and induce neurogenesis. Further, mechanisms at other levels (cellular and molecular) provide additional insight into how these exercise modalities influence memory. In Figure 2, we propose a hypothetical model suggesting that aerobic and resistance exercise may influence episodic memory via similar and unique mechanisms. Some of these differential mechanisms, which are detailed in the narrative that follows, includes modality-dependent alterations in neuroelectrical potentials and neuron-to-neuron communication.

### 5.1. Neuroelectrical Parameters

**Figure 3:**
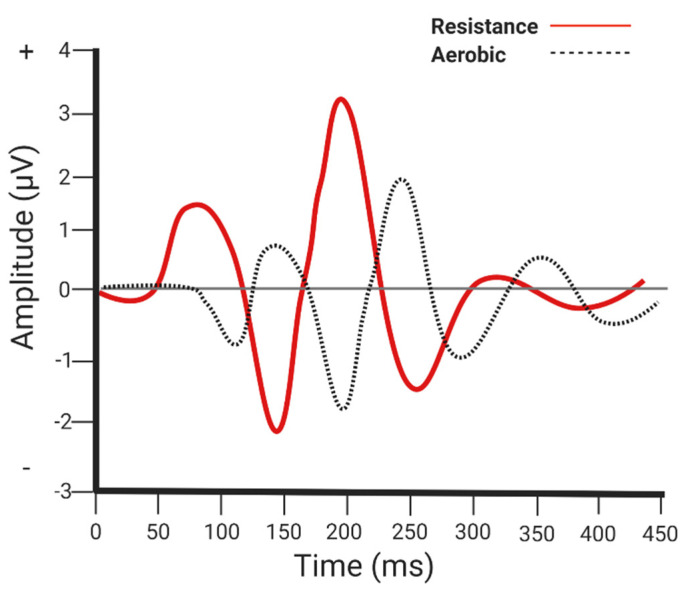
Neuroelectrical parameters. Aerobic and resistance exercise both improve event-related potential parameters of memory function; however, resistance exercise produces a shorter latency and greater amplitude of various event related potentials compared to aerobic exercise.

Ozkaya et al. [42] evaluated neuroelectrical correlates of memory function among those engaging in an aerobic (70% of heart rate reserve, 3 days/week, up to 50 min/session) vs. resistance (one to three sets of 12 repetitions of seven exercises) exercise program (9-weeks). One repetition maximum was estimated prior the first week of training by incorporating a four-repetition submaximal test to predict the one repetition maximum. Resistance training began at 60% of the estimated one repetition maximum and gradually increased by 5% every two weeks until participants were able to lift 80% of their estimated one repetition maximum. Both interventions improved various event-related potential parameters (e.g., electrophysiological brain responses to stimuli), but the resistance training group produced a shorter latency for P2/N2 and a larger amplitude for N1-P2, P2-N2, and N2-P3 (event-related potentials) (see Figure 3 for an illustration). These findings suggest that, perhaps, resistance training may have a slight differential influence on attentional resource allocation, which may help to optimize information processing and memory encoding [43,44].

### 5.2. Long-Term Potentiation and Related Parameters

An important potential cellular mechanism of memory is long-term potentiation (LTP) [45], or sustained excitatory post-synaptic potentiation. Stated differently, LTP involves persistent strengthening of synaptic connections occurring from high-frequency pre-synaptic activity. Episodic memories are considered to be stored in spatiotemporal patterns of neurons [46], and the greater the degree of communication among these neurons, the more enhanced the ability to store, access and retrieve the memory [47]. Various proteins (e.g., BDNF, CREB, IGF-1, β-CaMKII, PSD-95, PKCα, IL-6) and receptors (e.g., NMDA, TrkB) play a key role in influencing LTP. See Table 1 for a description of how these proteins and receptors influence memory. The narrative that follows discusses the potential unique influence of aerobic and resistance exercise on these proteins, receptors, and ultimately, episodic memory; see Figure 2 for an illustration depicting the exercise modality-specific effects on these proteins and receptors. Unless otherwise stated, these findings are derived from animal-based models.

Cytokines can alter synaptic properties, and thus, influence LTP [56,57]. For example, exogenous application (delivered in vitro to rat hippocampal slices) of IL-6 has been shown to inhibit LTP [58]. In humans with type 2 diabetes, chronic (12-weeks) resistance exercise, but not aerobic exercise, reduced levels of IL-6; however, there were no statistically significant differences in IL-6 between exercise modalities [59]. Other proteins also play an important role in subserving LTP [60,61,62], likely via alterations in key membrane receptors (e.g., NMDA) [63] and activating downstream cellular pathways (e.g., CREB; cAMP response element-binding protein) that influence late-phase LTP [64]. Circulating levels of IGF-1 (insulin-like growth factor-1; originating from the muscle) can pass the blood–brain-barrier, and thus, may centrally mediate actions of resistance exercise on memory function [65,66,67]. It is possible that resistance exercise (vs. aerobic exercise) may have a greater effect on peripheral IGF-1 production, whereas aerobic exercise may have a greater effect on increasing BDNF (brain-derived neurotrophic factor) production [68,69,70,71,72,73,74,75,76,77,78]. Importantly, both aerobic and resistance exercise have been shown to increase BDNF concentrations/expression [79,80], which may be intensity-dependent [78,81]. However, research has yielded inconsistent results on the IGF-1 response to exercise [82]. Elevated levels of blood-borne IGF-1 from running in rats has been shown to facilitate increased neuronal firing, c-Fos expression and BDNF production [83].

Additional insights have come from Cassilhas et al. [84,85] and others [86,87,88]. Cassilhas et al. [84] subjected rats to 8-weeks of aerobic treadmill training (30 min/day) or resistance exercise (ladder climbing for 20–30 min); control and sham groups were also included. Memory function was assessed from the Morris water maze task. Both training modalities improved learning and spatial memory in a similar manner. Similarly, both training modalities increased synapsin 1 and synaptophysin expression, which are essential proteins for neurotransmitter release and vesicle fusion. However, there was differential molecular signaling based on the exercise modality. The aerobic training increased hippocampal levels of IGF-1, BDNF, TrkB (tropomyosin receptor kinase B), and β-CaMKII (calmodulin-dependent protein kinase II), whereas the resistance training group increased peripheral and hippocampal levels of IGF-1, with concomitant activation of IGF-1 receptor and AKT in the hippocampus [84]. Recent work by Vilela et al. [87] also demonstrates similar findings. That is, 8-weeks of aerobic or resistance training (50 min, 3–4 d/wk) both increased spatial memory and also increased CREB and BDNF. However, aerobic exercise increased glutamatergic proteins (NMDA receptor and PSD-95; N-methyl-D-aspartate, postsynaptic density protein 95) and decreased DNA (deoxyribonucleic acid) damage, whereas resistance training increased levels of PKCα (protein kinase C alpha). Experimental work by Lee et al. [86] showed that 4-weeks of wheel running with and without resistance improved spatial learning and memory as well as BDNF expression. However, and unlike wheel running without resistance, wheel running with resistance also increased TrkB and CREB protein levels. This latter finding aligns with work by Tang et al. [88] showing that 6-weeks of aerobic treadmill exercise and weight-bearing ladder climbing improved learning and memory, but TrkB and CREB levels were upregulated to a greater extent in the ladder climbing animals (diabetic rats). Collectively, some of these findings suggests that work levels (i.e., degree of energy expenditure during the task), rather than running distance, may have unique effects on exercise-induced molecular mechanisms of memory.

Ultimately, this animal work suggests similar end-point effects (i.e., improved learning and memory), but distinct pathways may be activated to elicit these outcomes. Activation of these distinct pathways may also have differential cognitive effects, as, for example, AKT (protein kinase B) is critical for angiogenesis, neurogenesis, and neuronal cell survival, whereas β-CaMKII may be essential for NMDA modulation and memory consolidation [84]. For additional insight on bridging animal and human studies to improve our understanding of the effects of exercise on memory, the reader is referred to other excellent reviews on this topic [89,90].

### 5.3. Other Potential Candidate Mechanisms

Other related modality-specific mechanisms include, for example, alterations in neuronal oscillations and oxidative parameters. Brain oscillations are suggested to facilitate encoding, storage and retrieval of information in neural circuits [91]. For example, repeated bursts of 5–10 Hz (theta frequency) are optimal in inducing hippocampal LTP [92], with this rhythm being the most common during locomotor activities that have been described as “voluntary,” “preparatory,” “orienting,” or “exploratory” [93]. However, the extent to which acute aerobic and resistance exercise elicit similar or different effects of neuronal oscillation needs further investigation. Notably, independent experiments have shown that treadmill running [94], as well as climbing activities in rats [95], elicits rhythmical slow neuronal activity (e.g., theta waves).

Regarding oxidative parameters, Feter et al. [96] randomly assigned rats into a sedentary, moderate-intensity continuous training, high-intensity interval training, resistance training, or a running-wheel group. Training lasted approximately 40 days; the resistance training group engaged in climbing activities, whereas the moderate-intensity continuous training group engaged in aerobic exercise lasting 20–60 min a day. Both the moderate-intensity and resistance training groups improved memory, and similarly, both groups showed reduced nitrite levels in the hippocampus when compared to the control group (high levels may have a toxic effect on neurons). However, aerobic exercise provided antioxidant protection of the hippocampus by increasing hippocampal catalase activity, whereas resistance exercise increased reactive oxygen species, which plays an important role in IGF-1 signaling [97].

## 6. Conclusions

In this paper, we provide support that acute and chronic aerobic and resistance exercise (independently and combined) can, potentially, improve memory function. Likewise, the exercise-induced effect on memory function as a result of the combined effect of aerobic and resistance exercise may be due to an additive effect activating similar and/or unique mechanisms specific to the exercise modality. This additive effect may, in part, be driven by neural adaptations typically associated with the incorporation of resistance training. Neural adaptations are mediated by factors responsible for the strengthened neural communication between neural entities (LTP), thus, enhancing episodic memory. For example, an increase in proteins such as BDNF, CREB, PKCα, and NMDA (receptor), or a decrease in inflammatory markers (i.e., IL-6) can influence LTP at different phases (early and late). Findings clearly show that exercise possesses a unique effect on the molecular mechanisms responsible for memory enhancement. Future research could evaluate if these findings hold when exposed to varying external loads. For example, utilizing bodyweight vs. low external loads vs. high external loads (due to neurotrophic and hormonal changes) may influence the activation of these exercise-induced pathways, therefore, producing different cognitive effects, if any. Furthermore, the use of varying external loads may influence the production of peripheral or hippocampal BDNF (gene expression), possibly moderating the effect of exercise on memory function. In closing, aerobic and resistance exercise appears to improve memory function by activating similar and unique mechanistic pathways. Research should continue to explore this novel line of inquiry, as this may aid in the development of exercise programs that can be applied to neurological and memory-related disorders.

## Figures and Tables

**Table 1:** Role of key proteins and receptors in influencing long-term potentiation.

Protein	Role in Influencing LTP	Does Blocking This Parameter Influence Memory?	References
BDNF	Facilitates function and structural changes at the synapse. Induces the transformation of E-LTP to L-LTP by, for example, activating PI3K/AKT (protein transcription) and ERK (regulates dendritic and spine morphology) pathways and phosphorylation of CREB.	Yes	[48]
CREB	L-LTP via transcription of regulatory proteins.	Yes	[49]
IGF-1	Phosphorylation of voltage-gated calcium channels (increasing calcium influx and neurotransmitter release) and activates PI3K-AKT pathway.	Yes	[50]
β-CaMKII	Phosphorylation of AMPA receptors and exocytosis of AMPA receptors.	Yes	[51]
PSD-95	Receptor (e.g., AMPA-R incorporation) and synapse stabilization.	Yes	[52]
PKC	LTP induction mechanisms: increased release of pre-synaptic neurotransmitters; closure of dendritic chloride conductances.	Yes	[53]
**Receptor**			
NMDA	Calcium influx, redistribution of AMPA receptors, downstream activation of proteins to maintain L-LTP.	Yes	[54]
TrkB	Activation of MAPK, PI3K, and PLCγ pathways. MAPK and PI3K pathways ultimately have effects on neuronal survival and protein transcription; PLCγ activation increases release of intracellular calcium.	Yes	[55]

AMPA, α-amino-3-hydroxy-5-methyl-4-isoxazolepropionic acid; β-CaMKII, Calcium/calmodulin-dependent protein kinase type II subunit *beta*; BDNF, brain-derived neurotrophic factor; CREB, cAMP response element-binding protein; E-LTP, early-phase long-term potentiation; IGF-1, Insulin-like growth factor-1; L-LTP, late-phase long-term potentiation; MAPK, mitogen-activated protein kinase; NMDA, N-Methyl-D-aspartate; PI3K, Phosphoinositide 3-kinase; PKC, protein kinase C; PLCy, Phospholipase C (Y domain); PSD-95, postsynaptic density protein 95; TrkB, Tropomyosin receptor kinase B.
